# Finding the Cause of Psychosis: A Challenging Case of Anti-NMDAR Encephalitis

**DOI:** 10.1155/2020/2074704

**Published:** 2020-10-13

**Authors:** Bhanu R. Sabbula, Shravya Yemmanur, Raghavendra Sanivarapu, Deepthi Kagolanu, Ahmed Shadab

**Affiliations:** ^1^Department of Internal Medicine, Nassau University Medical Center, East Meadow, NY, USA; ^2^Division of Infectious Disease, Nassau University Medical Center, East Meadow, NY, USA

## Abstract

An autoimmune response causing inflammation in the brain tissue is called autoimmune encephalitis. Autoantibodies directed against N-methyl-D-aspartate (NMDA) receptors cause a type of autoimmune encephalitis resulting in memory loss, confusion, and psychosis. A 28-year-old male with a history of schizophrenia, seizure disorder, and stroke presented with a 2-day history of bizarre behavior, restlessness, insomnia, agitation, and hallucinations. He was initially managed for acute psychosis without any improvement. Further workup for organic causes revealed positive NMDAR antibodies in both the cerebrospinal fluid and serum, confirming a diagnosis of autoimmune encephalitis. His condition later improved with steroids and intravenous immunoglobulins. This case signifies the importance of ruling out organic causes in patients with unexplained neuropsychiatric symptoms. NMDA encephalitis is more common in young females with underlying malignancies, especially ovarian teratomas. This case is unique, given the extremely rare occurrence of NMDA encephalitis in male patients without any malignancies.

## 1. Introduction

Autoimmune encephalitis is caused by autoantibodies against neuronal cell surface/synaptic proteins called N-methyl-D-aspartate receptors (NMDARs) [1]. NMDARs play a significant role in maintaining synaptic plasticity and memory. Anti-NMDAR encephalitis has been associated with multiple infectious agents, including *Mycoplasma pneumoniae*, herpes simplex virus, measles virus, mumps, and group-A hemolytic *Streptococcus* [2]. Here, we describe the case of a young male with a known diagnosis of schizophrenia, presenting with confusion and hallucinations warranting psychiatric management and subsequently found to have anti-NMDAR encephalitis.

## 2. Case Presentation

A 28-year-old male presented with a 2-day history of restlessness, bizarre behavior, confusion, and hallucinations. His past medical history was significant for schizophrenia, seizure disorder, and stroke with residual left-sided facial droop. There was no family history of psychiatric disorders. His family reported that he had no recent fever, chills, nausea, vomiting, runny nose, sore throat, cough, headaches, urinary symptoms, loss of appetite, or loss of weight. On physical examination, he was afebrile and hemodynamically stable. He was alert, oriented to person and place, but not to time. His fiancé reported that he had been pacing around his room, not sleeping, acting paranoid, and hearing voices. He was admitted to the psychiatry department for acute psychosis, where he was unsuccessfully managed with valproic acid, risperidone, and benztropine, with haloperidol and diphenhydramine as needed. A review of his chart revealed two past admissions for a similar presentation of acute psychosis, and he was discharged with outpatient psychiatry follow-up recommendation. The patient failed to follow-up as an outpatient, and no further workup was done at that time. His hospital course was complicated by worsening mental status, and he continued to be unresponsive to the abovementioned medications.

**Figure 1:**
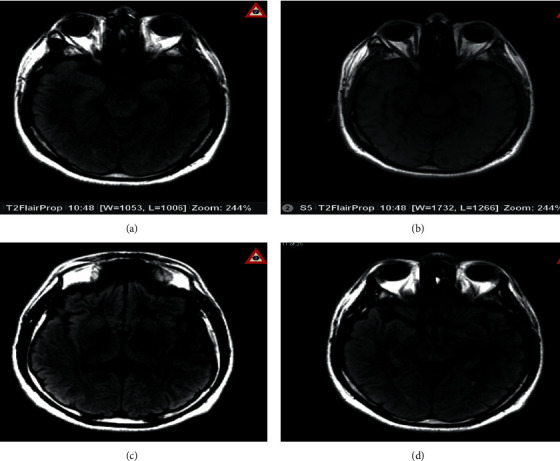
Axial magnetic resonance images (MRIs) demonstrating bright signal intensity and gyriform swelling in the region of the uncus (a) and thalami and insular cortex (b). This is best appreciated on flair images (c) and is harder to detect on T2-weighted images. The contrast study (d) does not show enhancement.

A thorough evaluation for organic causes was performed, including magnetic resonance imaging (MRI) of the brain with contrast, lumbar puncture (LP), and an autoimmune workup. The brain MRI with contrast showed multiple areas of bright signal intensity throughout the brain parenchyma, most notably in the medial temporal lobes and bilateral insular cortices, compatible with encephalitis (Figure 1). LP was performed, and cerebrospinal fluid (CSF) showed white blood cell count, 8 mm^3^; neutrophils, 1 mm^3^; lymphocytes, 95%; protein, 35 mg/dl; and glucose, 56 mg/dl. CSF testing for herpes simplex virus 1 and 2 DNA, varicella zoster PCR, VDRL, FTA ABS, enterovirus, and viral cultures were negative. A mild elevation of antinuclear antibody (1 : 40 titer) was noted. Blood and CSF were positive for oligoclonal bands.

Initially, the patient was started on empirical treatment with vancomycin, ceftriaxone, and acyclovir for possible infectious causes of encephalitis. The diagnosis of anti-NMDAR encephalitis was confirmed when the CSF was found positive for NMDAR antibodies. Empiric antibiotics and acyclovir were discontinued, and the patient was treated with intravenous immunoglobulin (IVIG) 400 mg/kg/day for 5 days and methylprednisolone 1 g/day for 5 days.

**Figure 2:**
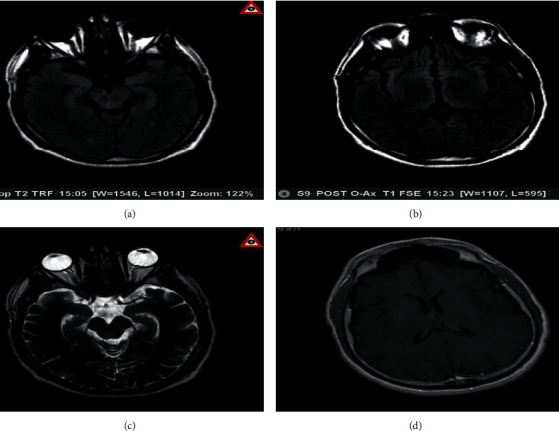
Axial magnetic resonance images (MRIs) one and a half weeks after treatment with immunoglobulin and methylprednisolone demonstrating gyriform swelling of the temporal lobes (a), especially medially, best seen on flair sequences (a, c, d). There is a faint bright signal intensity observed in the bilateral thalami and basal ganglia (c). Vague cortical brightness is also seen. There is no evidence of abnormal contrast enhancement (b). Compared to the earlier study, the degree of bright signal intensity is decreased, though swelling remains. These findings, with cortical, thalamic, and basal ganglia involvement, are typically seen in encephalitis.

The patient's condition improved significantly. He was observed to have more meaningful conversations and an improvement in his pressured speech. Repeat MRI after two weeks showed decreased bright signal intensity, although swelling remained the same (Figure 2). The patient was discharged and was followed up as an outpatient with complete resolution of his symptoms.

## 3. Discussion

Although this patient presented with features typical of anti-NMDAR encephalitis, his history of schizophrenia, seizures, and acute psychotic episodes contributed to delayed diagnosis. The classic presentation of this syndrome involves the development of psychiatric symptoms, memory loss, sleep disturbances, and seizures [3]. Malignancy is a known risk factor, and teratomas, germ cell tumors of the testes, and small cell lung cancer are specifically hypothesized to be associated with the disease [4, 5]. The presence of anti-NMDAR antibodies is specific for diagnosis. Although it has been associated with pathogens including *Mycoplasma pneumoniae*, herpes simplex virus, measles virus, mumps, and group-A hemolytic *Streptococcus*, these were all negative in our patient. The improvement of symptoms in our patient after treatment with IVIG and methylprednisolone further strengthens the diagnosis. The pathophysiology behind psychiatric manifestations of this disease is anti-NMDAR antibody inhibition of gamma-aminobutyric acid at the presynaptic junction in the thalamus and frontal cortex. Subsequently, postsynaptic glutaminergic neurons are uninhibited, resulting in dysregulation of the dopaminergic pathway [6].

Diagnosis of probable anti-NMDAR encephalitis requires the onset of at least four of the following symptoms within 3 months: abnormal behavior or cognitive dysfunction, speech dysfunction, movement disorder, dyskinesia, rigidity/abnormal posture, decreased level of consciousness, autonomic dysfunction, or central hypoventilation. Patients must also have either an abnormal electroencephalogram or CSF with pleocytosis or oligoclonal bands and reasonable exclusion of other disorders [7].

For a definitive diagnosis of anti-NMDAR encephalitis, IgG anti-GluN1 antibodies (NMDA receptor antibodies) should be positive in the presence of one or more of the six major groups of symptoms, after exclusion of other disorders [8]. Our patient met the diagnostic criteria for definitive anti-NMDAR encephalitis. The treatment approach to anti-NMDAR encephalitis includes removal of the etiological agents such as a tumor or treatment of the underlying infectious source and immunotherapy. The immunotherapies commonly used are corticosteroids, intravenous immunoglobulins, and plasma exchange with plasmapheresis in severe cases [8]. In resistant cases, immunosuppression is induced using rituximab, cyclophosphamide, mycophenolate mofetil, azathioprine, and methotrexate. Our patient responded well to immunotherapy with methylprednisolone and IVIG. Although symptoms are usually severe, studies suggest that patients are usually highly responsive to therapy [9]. As per a reported case series of 99 patients with all-cause encephalitis, 9.1% were found to have anti-NMDAR encephalitis. The mean age of these patients was 28 years, and 5 of 9 were females. Follow-up of these patients showed zero mortality and complete symptomatic recovery after treatment with high-dose corticosteroids and intravenous immunoglobulin [9]. Prompt diagnosis and early treatment will reduce mortality and ensure complete recovery.

In conclusion, anti-NMDAR encephalitis is a very rare entity in male patients, especially in those without any underlying malignancies. However, physicians should consider this diagnosis as a differential in any young patient presenting with unexplained neuropsychiatric symptoms. Younger patients who present with an initial episode of bizarre behavior should be assessed to rule out major organic causes, as delay in diagnosis leads to poor patient outcomes. Although literature suggests a predominance of anti-NMDAR encephalitis occurs in women with ovarian teratomas, this case demonstrates that the diagnosis should also be considered in men without any signs and symptoms of a neoplastic process, and a thorough workup for the infectious source should be conducted [2].

## Data Availability

The data used to support this study are restricted to protect patient privacy concern.
